# Novel multicellular organotypic models of normal and malignant breast: tools for dissecting the role of the microenvironment in breast cancer progression

**DOI:** 10.1186/bcr2218

**Published:** 2009-01-19

**Authors:** Deborah L Holliday, Kellie T Brouilette, Anja Markert, Linda A Gordon, J Louise Jones

**Affiliations:** 1 Centre for Tumour Biology, Institute of Cancer and CR-UK Clinical Centre, Bart's and The London, Queen Mary's School of Medicine and Dentistry, John Vane Science Centre, Charterhouse Square, London EC1M 6BQ, UK; 2 Prostate Cancer Research Centre, Department of Surgery, University College London, 3rd Floor Charles Bell House, 67 Riding House Street, London W1W 7EJ, UK

## Abstract

**Introduction:**

There is increasing recognition of the role of the microenvironment in the control of both normal and tumour cell behaviour. In the breast, myoepithelial cells and fibroblasts can influence tumour cell behaviour, with myoepithelial cells exhibiting a broad tumour-suppressor activity while fibroblasts frequently promote tumour growth and invasion. This study describes the development of physiologically relevant three-dimensional heterotypic culture systems containing mixed normal or tumour-derived breast populations and shows how such models can be used to dissect the interactions that influence cell behaviour.

**Methods:**

Populations of luminal cells, myoepithelial cells and fibroblasts were isolated from normal and malignant breast tissue, characterised and compared with immortalised cell lines. Co-localisation of normal and malignant luminal cells with myoepithelial cells alone or with either normal or tumour-derived fibroblasts was studied. Cultures were grown for seven days, and then gels were fixed and whole gel immunofluorescence carried out to assess co-localisation and polarisation. The potential role of matrix metalloproteinases (MMP) or hepatocyte growth factor(HGF)-c-met signalling in disrupting cellular organisation was investigated by incorporating inhibitors into cultures either alone or in combination.

**Results:**

Over a culture period of seven days, myoepithelial cells organised themselves around luminal cell populations forming dual-cell co-units. Characterisation of co-units showed established basal polarity and differentiation analogous to their *in vivo *counterparts. Tumour cell co-units revealed subtle differences to normal co-units including disruption of basement membrane and loss of β4-integrin, as described in ductal carcinoma *in situ *(DCIS) *in vivo*. Inclusion of normal fibroblasts had no influence on co-unit formation; however, inclusion of tumour-associated fibroblasts lead to disruption of co-unit organisation, and this was significantly inhibited in the presence of MMP and/or c-met inhibitors.

**Conclusions:**

To the best of the authors' knowledge, this study describes for the first time a co-culture model comprising three major components of normal and malignant breast: luminal cells, myoepithelial cells and stromal fibroblasts. These cells organise into structures recapitulating normal and DCIS breast, with homing of myoepithelial cells around the luminal population. Importantly, differences are exhibited between these systems reflecting those described in tissues, including a central role for tumour-associated fibroblasts and MMPs in mediating disruption of normal structures. These findings support the value of these models in dissecting normal and tumour cell behaviour in an appropriate microenvironment.

## Introduction

Over the past decade the importance of the microenvironmental control of tumour cell progression has been increasingly recognised. The microenvironment within the breast is complex, consisting of a stromal component, the major cell type of which is the fibroblast along with inflammatory cells and blood vessels. In addition, there is a non-neoplastic epithelial component in the myoepithelial cell that lies between the luminal cell layer and the basement membrane. Both of these cell types are known to influence tumour progression; tumour-associated fibroblasts (TAFs) have been shown to promote tumour cell invasion [1-3], release extracellular matrix (ECM) degrading proteases [1,4,5] and modify the composition of the ECM facilitating tumour cell motility [6]. In contrast, myoepithelial cells which form a barrier between tumour cells and the surrounding stroma are believed to play a tumour-suppressing role. This could in part be due to the ability of myoepithelial cells to decrease tumour cell proliferation and increase apoptosis, and to reduce tumour cell invasion and protease expression *in vitr*o [7,8].

The exact role of these cell types, interacting both with each other and with the tumour cell, in the progression of breast cancer has yet to be fully understood; however, it is likely that the influence of each of these cell types differs during the stages of breast cancer, for example, myoepithelial cells are present in ductal carcinoma *in situ *(DCIS) but are lost in the progression to invasive carcinoma. Furthermore, the function of the cells of the microenvironment may change during evolution of the tumour, because genetic and phenotypic differences have been identified in these populations in tumour tissues compared with normal tissues [6,9]

Over recent years there has been a shift towards examining cells in physiologically relevant matrices that are able to more faithfully recapitulate the multi-cell three-dimensional (3D) environment of breast carcinomas *in vivo *[10]. Culturing cells in 3D has been shown to have dramatic effects on cell polarity and differentiation as well as signalling cascades and gene expression profiles compared with that seen in monolayer culture [11-13]. Studies of mammary epithelial cells grown in the basement membrane equivalent, Matrigel, have allowed a deeper understanding of mammary gland development and in particular the key roles played by molecules such as the integrins and laminin in maintaining tissue architecture and cell polarity in the normal breast [12,14]. Furthermore this approach has permitted the identification of proteins or receptors which are altered in cancer, such as up-regulation of β1-integrin, with reversion to a normal phenotype when the actions of this integrin are blocked [13].

Fibroblasts have been more widely studied in 3D, most frequently in a collagen matrix, which is more representative of their physiological stromal surrounding [14,15]. As well as being morphologically very different in 3D because of the loss of the enforced polarity seen in 2D cultures, they form novel matrix adhesions with the ECM rather than fibrillar or focal adhesions seen in monolayers [15]. Thus modelling of tumour biology is cell-type and stroma-context dependent, and alterations of these from a 2D to a more physiological 3D system produces striking changes.

Although current 3D models of breast cancer are a considerable improvement on 2D systems, they are limited in focussing on the behaviour of one cell type in 3D or, at best, on the interactions between two cell types [16-18]. The complex nature of the stromal component in the breast means that the study of one or two cell types is unlikely to recapitulate what is seen *in vivo*. For example, the balance between the tumour suppressor role of myoepithelial cells and the pro-invasive role of fibroblast populations cannot be gauged in a two-cell system.

To our knowledge, no published model has included all three cell populations in one system. This study describes a reproducible system developed to co-culture normal or malignant epithelial cells, myoepithelial cells and fibroblast populations in an appropriate ECM, validates the architectural and functional recapitulation of the *in vivo *phenotype, and demonstrates the application of such a model in dissecting cellular interactions. This provides a physiologically relevant *in vitro *model system to study the role of the microenvironment in the normal and malignant breast.

## Materials and methods

### Breast tissue and cell lines

Breast tissue was obtained from women undergoing surgery for breast carcinoma or reduction mammoplasty after informed consent was given and the study was approved by the North East London Ethics Committee. None of the patients had received pre-operative chemotherapy.

The breast cancer cell line, MCF-7, and the human fetal foreskin fibroblast cell line, hfff_2_, were obtained from the American Type Culture Collection (Rockville, Maryland). The myoepithelial (MYO1089), luminal epithelial (HB4a) and fibroblast (HMFU19) cell lines were a gift from Professor Mike O'Hare (Ludwig Institute, London, UK). These lines were generated from isolated primary cell populations by transduction with SV40 large T antigen [19].

### Isolation of primary cell populations

Tissue, excess to histopathological diagnosis, was selected from the breast samples and fibroblasts were isolated as described previously [20]. Briefly, the tissue was digested for 12 hours in Dulbecco's modified eagle's media (DMEM), 10% fetal bovine serum (FBS), 2MM L-Glutamine, 100 IU penicillin and streptomycin, 400 IU collagenase IA and 65IU hyaluronidase (all reagents obtained from Sigma-Aldrich Company Ltd., Poole, Dorset, UK). Following a sedimentation step at 1g for 30 minutes, the supernatant was removed from the organoid portion and centrifuged, washed twice in serum-free DMEM then filtered through a 20 μm cell strainer (BD Falcon, San Diego, California, USA). The filtrate was spun down and the cell pellet resuspended in DMEM with 10% FBS, penicillin and streptomycin (100 U/ml) and fungizone (2.5 μg/ml) (GiboBRL, Invitrogen, Corporation, Carlsbad, CA, USA). The cells were cultured for 24 hours then the medium was aspirated off and the cells were washed with DMEM, (to remove any non-viable cells or contaminating red blood cells) before re-feeding with complete DMEM.

The residual organoids were washed twice in serum-free DMEM (Sigma-Aldrich Company Ltd., Poole, Dorset, UK) and plated into either luminal media (RPMI 1640, Sigma-Aldrich Company Ltd., Poole, Dorset, UK) or myoepithelial media (Hams F12 Nutrient mix, Sigma-Aldrich Company Ltd., Poole, Dorset, UK) and cultured for four to seven days until cells could be observed growing out from the organoids. Cells were then trypsinised and resuspended in PBS/0.1% BSA containing immunomagnetic beads labelled with β4-integrin or epithelial membrane antigen (EMA) to isolate myoepithelial and luminal cells, respectively as previously described [21]. The resulting cell populations were cultured in cell specific media [20] and used within two passages.

### Characterisation of cell populations

Cells plated onto poly-l-lysine coated coverslips were stained with antibodies to vimentin (clone 384, Dako UK Ltd, Ely, UK), Cytokeratin 14 (clone LL002, AbD Serotec, MorphoSys, Kidlington, UK), Cytokeratin 18 (clone CY90, AbD Serotec, MorphoSys, Kidlington, UK), CK17 (clone CK-E3, Sigma-Aldrich Company Ltd., Poole, Dorset, UK), CK5/6 (clone D5/16 B4, Dako UK Ltd, Ely, UK), EMA (clone E29, Dako UK Ltd, Ely, UK), α-smooth muscle actin (α–smooth muscle actin (SMA), clone 1A4 Dako UK Ltd, Ely, UK), E-cadherin (NCH-38, Dako UK Ltd, Ely, UK), DSG-2 (Progen), DSG-3 (clone 5H10, Autogen Bioclear, Calne, UK), β4 integrin (clone 439-9B, Chemicon, Millipore, Watford, UK), CD45 (clone 2B11/PD7/26, Dako UK Ltd, Ely, UK) and CD31 (clone JC70A, Dako UK Ltd, Ely, UK). Fluorescein isothiocyanate-conjugated rabbit anti-mouse F(ab^1^)_2 _(Dako UK Ltd, Ely, UK) was used as secondary and 4',6-diamidino-2-phenylindole (DAPI) (Calbiochem) as a nuclear counterstain. Omission of the primary antibody was included as a negative control. Images were recorded on a Zeiss Axiovert 200M confocal microscope using the LSM510 Meta software (Carl Zeiss, Jena, Germany).

### Construction of heterotypic 3D collagen gel cultures

Myoepithelial cells and fibroblasts were labelled with 5 μg/ml cell tracker (Cell tracker red *CMTMR *or Cell tracker green *CMFTA *respectively, Molecular Probes Invitrogen, Corporation, Carlsbad, CA, USA) 24 hours before use.

Luminal cells (3.3 × 10^5^) were resuspended in 2 mls of DMEM and aggregated over agar for two hours at 37°C / 5% carbon dioxide. An equal number of myoepithelial cells labelled with red cell tracker was then added to the luminal population and cells aggregated for a further two hours. Aggregates were centrifuged gently at 500 rpm for three minutes and resuspended in 1 ml of collagen with or without fibroblasts.

Rat tail collagen I (BD Biosciences, New Jersey, USA) was adjusted to a concentration of 1 mg/ml by diluting with Dulbecco's PBS. Eight parts of collagen were then mixed with one part 10 × Hank's Buffered Salt Solution (Sigma-Aldrich Company Ltd., Poole, Dorset, UK). Sodium hydroxide was added drop-wise to neutralise the pH (judged by eye when the mixture turned from yellow to orange-red). One volume of DMEM with or without 3.3 × 10^5^/ml fibroblasts was added and mixed into the gel by gentle pipetting. Into each 10 mm diameter MatTek dish (MatTek Corporation, US) 100 μl of the gel mixture was pipetted and left to solidify for 10 minutes at 37°C / 5% carbon dioxide before being covered with 1.5 ml complete DMEM. Gels were cultured for seven days, media was replaced every two to three days during this time. Following optimisation, selected cultures were carried out using animal-free reagents.

For experiments investigating formation of co-units, a time course experiment was carried out whereby 3.3 × 10^5 ^cells of each population were mixed together as a single cell suspension without pre-aggregation of individual populations. The admixed cells were resuspended in collagen and cultured as described above and cells were fixed at day 1 (D1), D3, D5 and D7.

Experiments including inhibitors to c-met and matrix metalloproteinases (MMP) were set up as for the time course experiment (n = 3 for each condition). A selective inhibitor for the hepatocyte growth factor (HGF) receptor, c-met, (100 nM, PHA 665752, Tocris) and/or a broad spectrum MMP inhibitor (10 μM, GM6001, Chemicon, Millipore, Watford, UK) was included in the media at D1 with additional inhibitor added at D3 and D5. Cultures containing a vehicle control of 10 μM dimethyl sulfoxide (DMSO) (Sigma-Aldrich Company Ltd., Poole, Dorset, UK) were also included.

### Generation of fibroblast conditioned media

Fibroblasts were plated into 25 cm^2 ^tissue culture flasks and grown to 60% confluency, the media removed, cells washed with Dulbecco's PBS and 5 ml serum-free DMEM was added. Fibroblasts were then cultured for a further 48 hours, media removed and centrifuged to remove cell debris and stored at -80°C until use.

### Fixation of gels and whole gel immunofluoresence

Gels were washed in PBS (three times for five minutes), fixed in 4% paraformaldehyde (Fisher Scientific, Loughborough, UK) for 30 minutes at room temperature and then permeabilised with 0.5% triton X-100 (Sigma-Aldrich, Company Ltd., Poole, Dorset, UK) in PBS for 30 minutes at room temperature. Immunofluorescence was carried out on whole gels contained within the MatTek dishes. Non-specific binding was blocked with normal goat serum for 10 minutes before incubation with a primary antibody, E-cadherin, EMA, Tenascin-C (TN-C; Clone BC24, Sigma-Aldrich Company Ltd., Poole, Dorset, UK) and β4-integrin for 90 minutes at room temperature. Secondary antibody (goat anti-mouse Alexa 488) was incubated for 30 minutes at room temperature along with the nuclear stain DAPI and gels were gently compressed between the dish and a coverslip. Images were captured on the confocal microscope.

### Assessment of co-unit formation and basal polarity

In experiments assessing co-unit formation the number of co-units formed (defined as an aggregate where at least 70% of the luminal (or MCF-7) cells are surrounded by myoepithelial cells) in 10 microscopic fields was counted and an average of co-units/10 fields calculated.

For assessment of basal polarity the number of co-units expressing β4 integrin and TN-C (basement membrane deposition) was counted in 10 fields and data expressed as a percentage of the total co-units.

## Results

### Isolation and characterisation of primary breast cell populations and cell lines

Fibroblast, luminal epithelial and myoepithelial cell populations were isolated from reduction mammoplasty material (n = 12) and breast cancer tissue removed (n = 12). In the tumour donor cases, the fibroblasts were isolated from within the carcinoma (i.e. TAFs).

**Figure 1:**
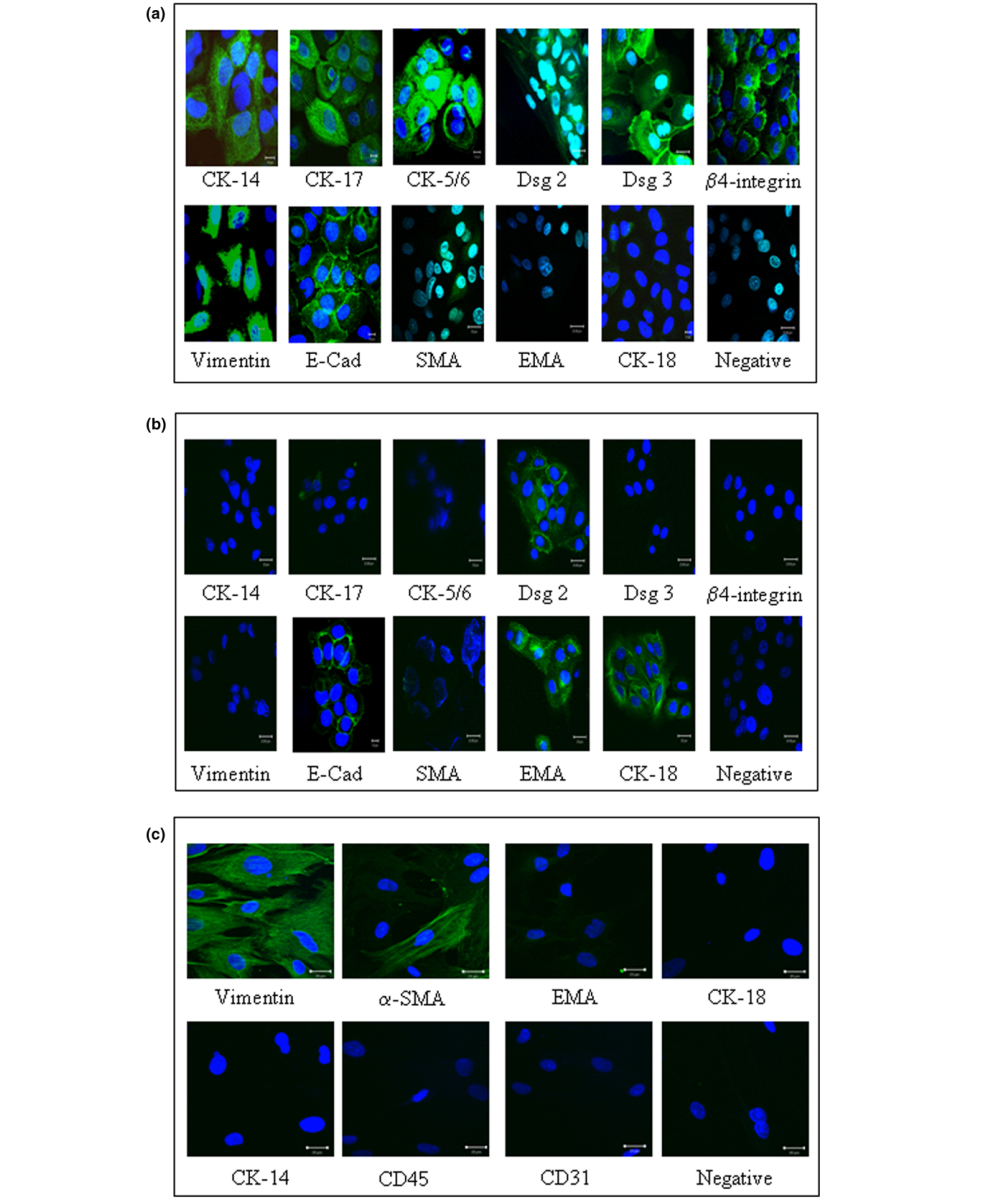
Characterisation of cell populations. The purity and phenotypic integrity of isolated primary and immortalised cell populations was confirmed by immunofluorescence. **(a)** Primary myoepithelial cells expressed of CK-14, CK-17, CK5/6, desmoglein (Dsg) 2, Dsg3, β4-integrin, vimentin, E-cadherin (E-Cad) and weak expression of α-smooth muscle actin (SMA). No expression of the luminal associated epithelial membrane antigen (EMA) and CK-18 was observed. **(b)** Isolated luminal cells expressed EMA and CK-18 along with expression of Dsg-2 and E-cadherin. **(c)** Isolated fibroblasts expression vimentin and a proportion of the cells expressed α-SMA but there was no expression of epithelial, macrophage of endothelial associated markers. The immortalised cell lines HB4a (luminal), MYO1089 (myoepithelial), HMFU19 (normal fibroblast) and hfff2 (fetal fibroblast) exhibited identical patterns of marker expression in comparison to their primary counterparts (data not shown).

The phenotype of different cell populations and an estimate of purity was established by immunostaining. Primary myoepithelial cells showed expression of basal-associated cytokeratins-14, -17 and -5/6 and the myoepithelial associated β4-integrin (Figure 1a). The myoepithelial-restricted desmoglein-3 was expressed along with desmoglein-2 and E-cadherin. Myoepithelial cells showed characteristic expression of the mesenchymal marker vimentin but were negative for the luminal epithelial-associated EMA and CK18. There was a down-regulation of α-SMA expression in the isolated myoepithelial cells compared with their tissue counterparts. The myoepithelial cell line MYO1089 exhibited an identical expression profile as primary myoepithelial cells (data not shown).

Isolated luminal cells showed expression of the luminal cytokeratin 18 and the luminal epithelial-associated EMA, with membranous staining for desmoglein-2 and E-cadherin. The cells were negative for the myoepithelial and fibroblast markers (Figure 1b). The luminal cell line HB4a showed the same expression profile as the primary luminal cells (data not shown).

The isolated fibroblasts stained uniformly for vimentin and were negative for the luminal epithelial and myoepithelial markers, the endothelial-associated CD31 and the pericyte and vascular smooth muscle-related alpha1-integrin (Figure 1c). The fibroblasts were also negative for the leucocyte marker CD45 (not shown). A proportion of the fibroblasts in any given isolate were positive for α-SMA (about 20%) and this was comparable between normal and TAF's. The fibroblast cell lines HMFU19 and hfff2 showed the same expression pattern as primary cells (data not shown).

### Luminal and myoepithelial cells form dual-cell co-units

**Figure 2:**
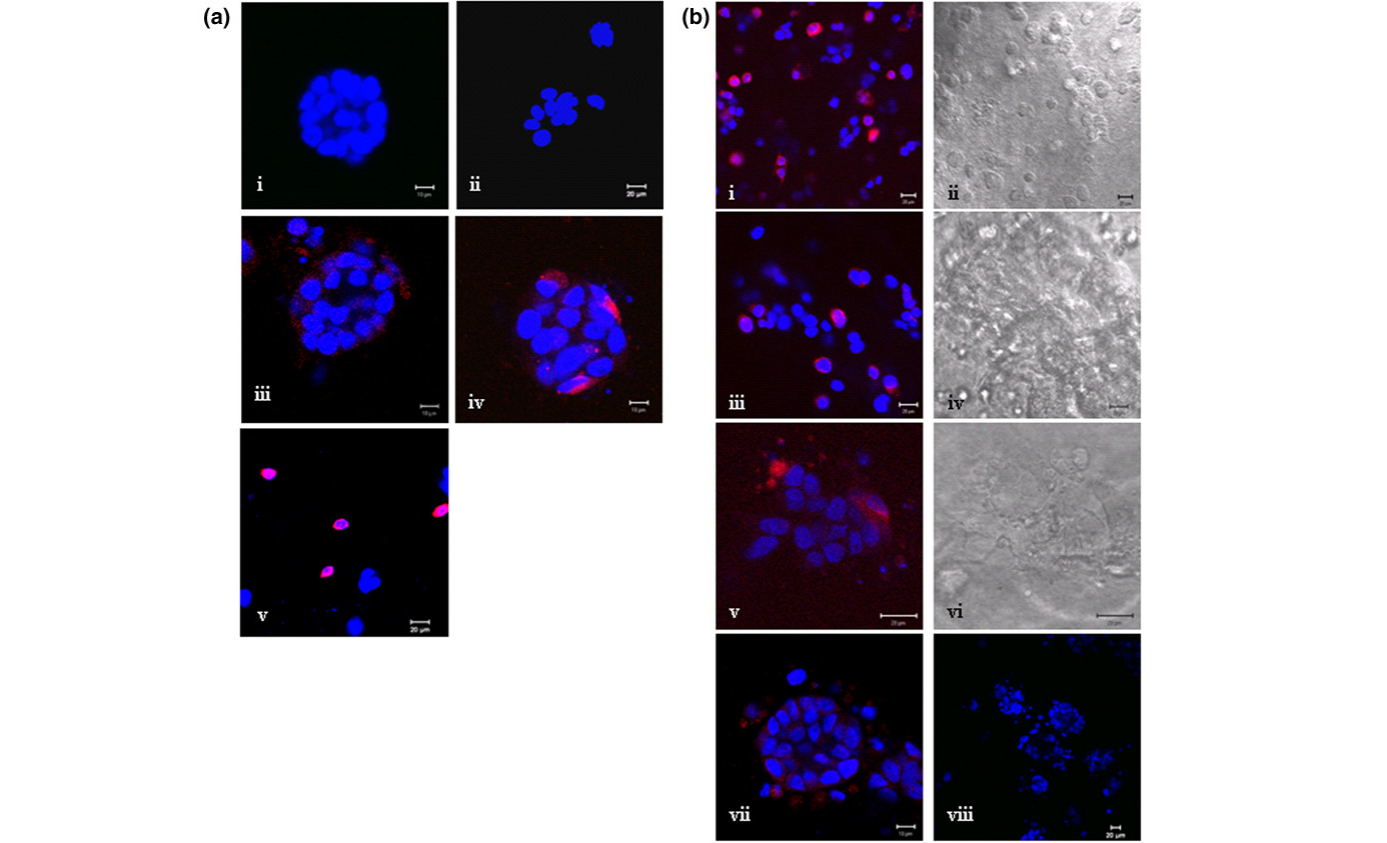
Generation of epithelial-myoepithelial co-units. Cell populations were cultured in collagen gels for seven days with preaggregation of the luminal cell population. **(a) **(a)i MCF-7 cells alone formed tight spheres whereas (a)ii luminal cells formed loosely cohesive structures. Co-culture of (a)iii primary luminal/myoepithelial cells and (a)iv MCF-7/myoepithelial cells showed organisation of the myoepithelial cells (cell trackered in red) around the luminal population (nuclear labelling blue with 4',6-diamidino-2-phenylindole (DAPI)) forming a dual-cell co-unit. Co-unit formation was shown to be myoepithelial specific by the lack of organisation of (a)v luminal cells (cell trackered red) around pre-aggregated MCF-7 cells. A time course to show co-unit formation from cells without prior preaggregation was performed. **(b) **At (b)i,ii day 1 and (b)iii,iv day 3 MCF-7 cells and primary myoepithelial cells were intermingled. At (b)v,vi day 5 cells began to aggregate and by (b)vii,viii day 7 co-units were formed suggesting that organisation of co-units is through an active homing process.

After seven days in culture, collagen gels were harvested to examine structural morphology. MCF-7 cells alone formed tight spheres within the gel of about 30 cells in size (Figure 2ai), whereas normal luminal cells (both primary and HB4a) grew primarily as single cells or small loosely cohesive groups (Figure 2aii). Inclusion of myoepithelial cells with either normal or malignant luminal cell populations resulted in organisation of the myoepithelial cells around the luminal population to form a dual-cell co-unit (Figure 2aiii,iv). This organisation was shown to be myoepithelial specific because co-culture of the two luminal populations did not result in similar co-aggregation (Figure 2av).

To investigate whether the organisation of these co-units was an active homing process of myoepithelial cells around the luminal population, the two cell populations were mixed at the start of the experiment with no aggregation before resuspension in collagen. Gels were harvested at several time points to assess the development of co-units. On D1 (Figure 2bi,ii) and D3 (Figure 2biii,iv), the two cell types are intermingled, but by D5 (Figure 2bv,vi) the cell populations began to aggregate and by D7 (Figure 2bvii,viii) the co-units were formed, with myoepithelial cells organised around the MCF-7 population indicating an active process of spatial organisation that we term 'homing'. Even by D7 no lumen formation was evident in any of the cultures.

### Phenotypic analysis of co-units

To analyse the structural and functional characteristics of the dual-cell co-units, whole gel immunofluorescence was used to examine a series of key markers. In all cases, a similar pattern of expression was seen with primary cell populations and their immortalised counterparts.

**Figure 3:**
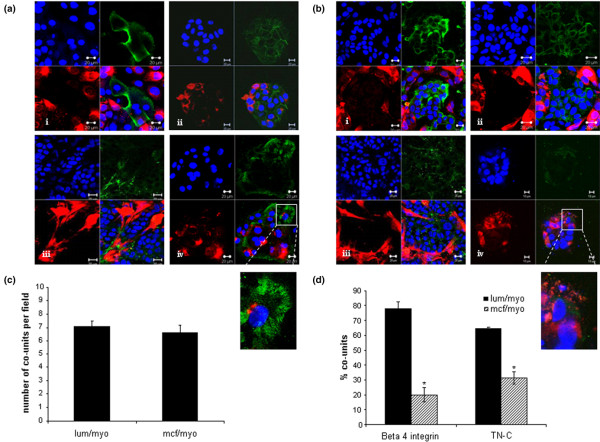
Phenotypic analysis of co-units. **(a) **Co-units formed by primary myoepithelial cells (red) surrounding the normal luminal epithelial cells exhibited polarity detected by luminal expression of epithelial membrane antigen (EMA) localised to (a)i, green stain: the epithelial population. (a)ii, green stain: E-cadherin was expressed at cell-cell junctions. (a)iii, green stain: Myoepithelial cells laid down the matrix protein tenascin-C (TN-C) and (a)iv, green stain: expressed β4-integrin in hemidesmosome like structures at the myoepithelial-gel interface. **(b) **In the myoepithelial/MCF-7 co-units, (b)i, green stain: MCF-7 tumour cells expressed EMA and (b)ii, green stain: E-cadherin. A less organised basement membrane was present, (b)iii, green stain: shown by TN-C staining and (b)iv, green stain: marked down-regulation or loss of β4-integrin expression was observed. Quantitation of co-unit formation was carried by counting the number of co-units in 10 microscopic fields per culture to give an average number of co-units/field. A co-unit was defined as aggregated luminal or MCF-7 cells which were at least 70% enclosed by myoepithelial cells. **(c) **No significant difference in the two types of co-units was observed. To quantitate the presence of β4-integrin and TN-C the percentage of co-units expressing the proteins were counted and expressed as a percentage of total co-units. **(d) **A significant decrease in the levels of β4 integrin and TN-C were observed in the MCF-7-myoepithelial cell cultures compared with the luminal-myoepithelial cell cultures. (p = 0.001).

In the model of 'normal' breast, there was strong expression of EMA limited to the central luminal HB4a population (Figure 3ai). E-cadherin was also strongly expressed by the luminal cells (Figure 3aii). A zone of TN-C, localised to the myoepithelial-gel interface was evident, indicative of endogenous basement membrane production (Figure 3aiii). Similarly, strong basal polarised expression of β4-integrin was seen in the myoepithelial cells located in structures resembling hemidesmosomes (Figure 3aiv).

In the MCF-7-myoepithelial co-cultures, a similar pattern of myoepithelial homing around luminal population was observed. The tumour cells expressed EMA and E-cadherin expression was also maintained (Figure 3bi–ii); however, there were subtle differences in myoepithelial characteristics. Basement membrane was less organised, present as punctate deposits rather than a discrete layer around the myoepithelial cells (Figure 3biii). In addition there was marked down-regulation of β4-integrin expression, which was weakly expressed, or in some cases negative, in these tumour co-units (Figure 3biv).

These characteristics were consistent features in co-cultures generated from a minimum of six primary donors and 10 cell line cultures.

Quantitative analysis of normal (primary n = 4; cell lines n = 4) and MCF-7 (primary n = 3; cell lines n = 4) showed the average number of co-units per microscopic field was seven, with no significant difference in the number of co-units between primary and cell line cultures, or luminal-myoepithelial and MCF-7-myoepithelial co-cultures (Figure 3c). However, significant differences were seen in the expression of the basal polarisation marker, β4-integrin and the basement membrane protein TN-C. In luminal-myoepithelial co-units 78% of co-units expressed β4-integrin compared with 20% of the MCF-7-myoepithelial co-units (p ≤ 0.001, Figure 3d). A similar loss in TN-C was observed in the MCF-7-myoepithelial co-units with only 31% expressing the protein compared with 64% luminal-myoepithelial cultures (p ≤ 0.001, Figure 3d).

### Normal fibroblasts and tumour-associated fibroblasts have different effects on co-unit structure

To recapitulate the epithelial-stromal interactions present in the *in vivo *situation, cultures were constructed to incorporate a fibroblast population with the epithelial-myoepithelial co-unit.

**Figure 4:**
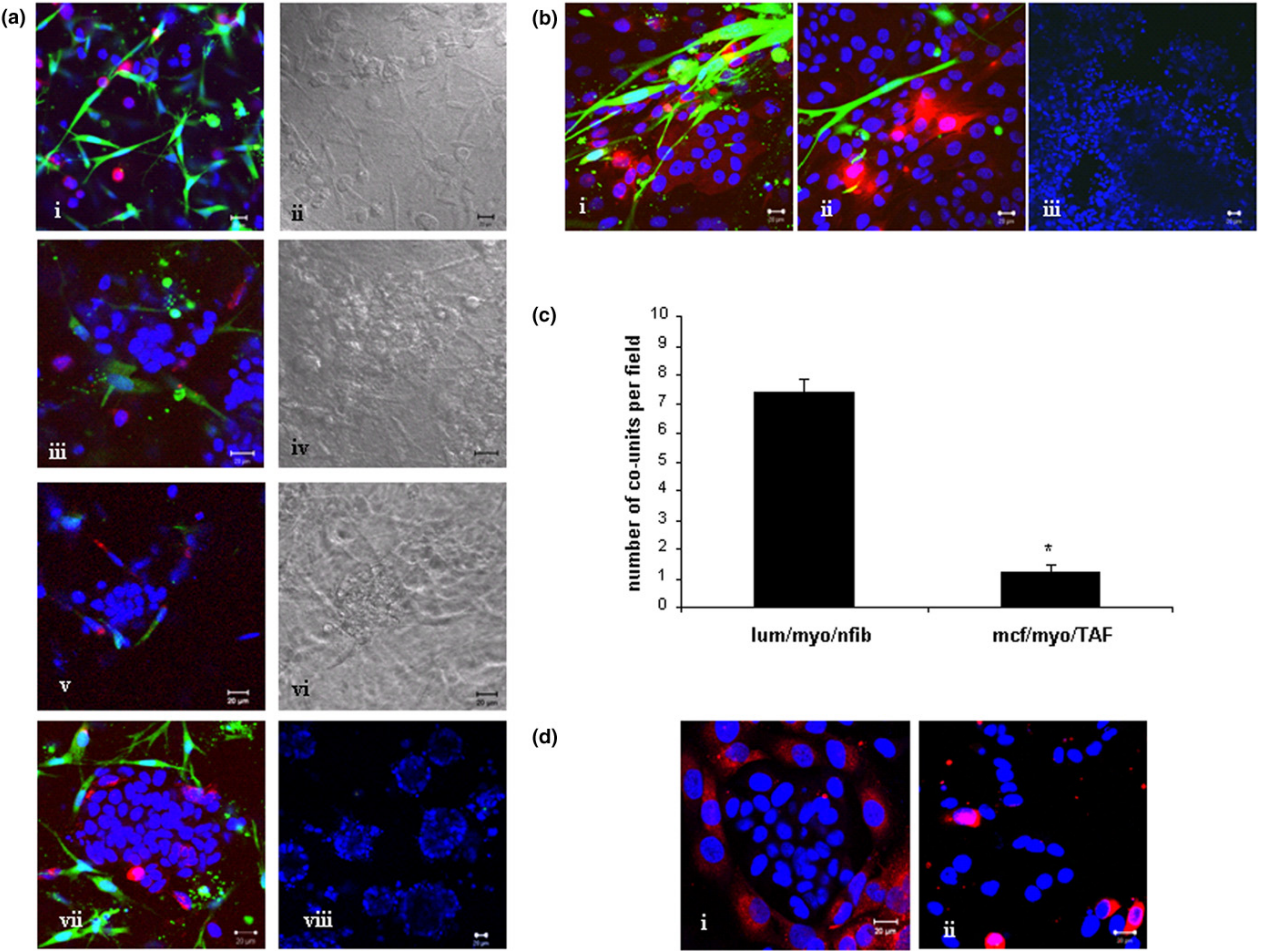
Effect of fibroblasts on co-unit structure. Incorporation of normal breast fibroblasts into myoepithelial/MCF-7 cultures. Non pre-aggregated cells were mixed and cultured in a collagen gel for up to one week to establish if fibroblasts influence epithelial co-unit formation. **(a) **(a)i,ii: At day 1, MCF-7 cells, myoepithelial cells (cell trackered red) and fibroblasts (cell trackered green) were intermingled. (a)iii,iv: By day 3, MCF-7 clusters were beginning to form and (a)v,vi these were more prominent by day 5. (a)vii,viii: Co-units were formed by day 7 with normal fibroblasts located around the epithelial structures (low power image). Incorporation of tumour-associated fibroblast (TAF) into myoepithelial/MCF-7 into 7 day co-cultures. **(b) **(b)i,ii: The homing of myoepithelial cells to MCF-7 cells is disrupted on addition of TAFs, with the cells reverting to an intermingled mixed population. (b)iii: The effect on the culture is particularly evident at low-power magnification. **(c) **Quantitation of co-units revealed a decrease from 7.5 to 1.2 in the presence of TAFs compared with normal fibroblasts (p = 0.001). **(d) **These effects could be replicated in cultures grown for seven days in the presence of conditioned medium from the fibroblast populations with disruption of co-unit architecture (d)ii in the presence of TAF conditioning media but not (d)i normal fibroblast conditioning media.

Incorporation of normal fibroblasts from reduction mammoplasty tissue, or the 'normal' fibroblast cell line HMFU19 revealed a similar pattern of co-unit formation to those cultures without a fibroblast population over the same time period. At D1 (Figure 4ai,ii) the three cell populations were intermingled, and on D3 (Figure 4aiii,iv) there was some clustering of luminal cells, which became more marked by D5 (Figure 4av,vi). At D7 myoepithelial cells had homed around the luminal cell clusters with fibroblasts located around the co-unit having no significant effect on the architecture of the co-unit structure (Figure 4avii,viii).

In contrast, addition of TAFs to the cultures led to disruption of the co-units, with loss of homing of the myoepithelial cells and mixing of all three cell populations (Figure 4b). Quantitative analysis of these cultures revealed that the average number of co-units per field is significantly decreased from 7.5 in the presence of the normal fibroblasts to 1.2 in the presence of TAFs (p ≤ 0.001, Figure 4c)

Experiments were performed where conditioned medium from normal fibroblasts or TAFs was added to the co-cultures in place of the fibroblast populations. The effect seen in the presence of the fibroblasts is replicated by their conditioned media, with TAF conditioned medium (Figure 4dii), but not with normal fibroblast conditioned medium (Figure 4di) disrupting epithelial co-units.

### Inhibiting HGF receptor and MMPs can restore epithelial co-unit formation

**Figure 5:**
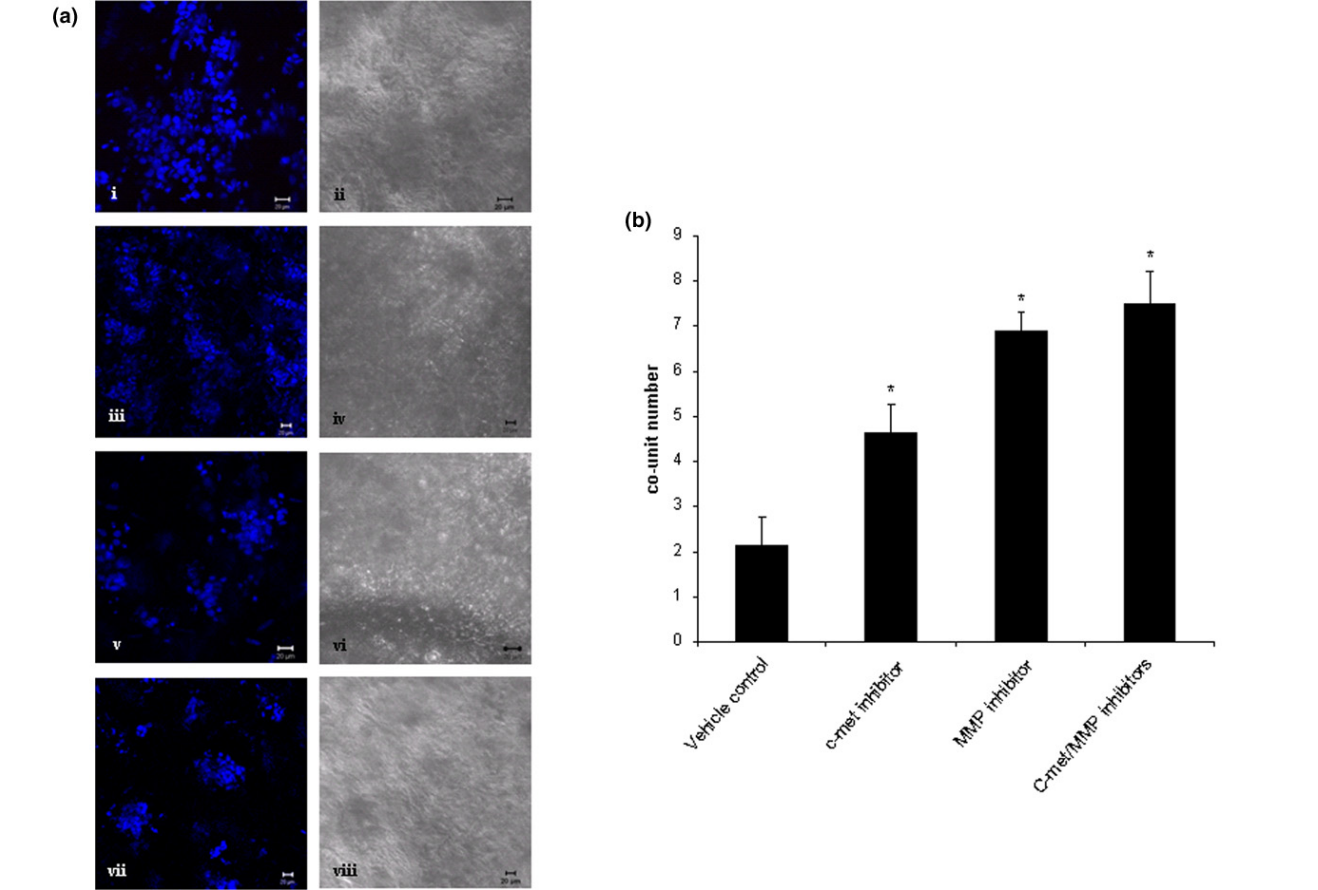
Effect of inhibitors on fibroblast-induced co-unit disruption. **(a) **Cultures were generated with MCF-7 cells, myoepithelial cells and tumour-associated fibroblasts (TAFs) without prior aggregation, and grown in the (a)i,ii presence of dimethyl sulfoxide (DMSO) (10 μM), a specific inhibitor for c-met, (a)iii,iv the receptor for HGF, (PHA 665752, 100 nM), (a)iv,vii a broad spectrum matrix metalloproteinase (MMP) inhibitor (10 μM) individually, or (a)vii,viii in combination. Inhibitors were replenished at two-day intervals and the cultures harvested at seven days. **(b) **In the vehicle control cultures exhibited an average of two co-units per microscopic field. This was significantly increased in the presence of the c-met inhibitor (average co-units 4.6, p = 0.016), the MMP inhibitor (average co-units 7, p ≤ 0.001) and the inhibitors in combination (average co-units 7.5, p = 0.001).

To investigate potential mechanisms by which TAFs may be disrupting co-unit formation a series of experiments was carried out to analyse the potential involvement of HGF-c-met interactions and MMP activity. Inhibitors to either the HGF receptor (c-met) and a broad spectrum MMP inhibitor were incorporated into MCF7/myoepithelial/TAF cultures, either alone or in combination, and the frequency of co-unit formation was assessed in comparison to vehicle-only controls (n = 3 for each condition). A greater number of co-units were formed in the presence of either inhibitor compared with the vehicle control (Figure 5a). The presence of the c-met inhibitor significantly increased the average number of co-units from two in the control group to 4.5 (p = 0.016). This effect was greater in the presence of the MMP inhibitor where an average of seven co-units formed (p = 0.001). A combination of the two inhibitors also resulted in significantly higher numbers of co-units than controls (p = 0.001) but this was not significantly greater than in the presence of MMP inhibitor alone (Figure 5b).

## Discussion

There is growing evidence for the role of the microenvironment in cancer progression. Fibroblasts clearly influence tumour behaviour [1-3] and, in the breast, myoepithelial cells also appear to be a major regulator of tumour progression [7,8].

Here, we describe for the first time, a 3D heterotypic co-culture model including two major cellular components of the breast microenvironment. This recapitulates the organisation and functional differentiation of the breast, and will provide a valuable tool to dissect the complex interactions between these cell populations in order to understand the factors determining breast cancer behaviour.

There is a paucity of well-characterised, validated cell lines to model the breast microenvironment; therefore, primary isolated populations remain the 'gold standard'. Thus, central to this study was the need to isolate pure populations of primary luminal, myoepithelial and fibroblast populations and ensure that their phenotype is maintained in culture. No single marker can reliably distinguish between these breast populations. For example, vimentin and α-SMA will label both myoepithelial and fibroblast populations, and while the cytokeratin profile is frequently used to differentiate luminal and myoepithelial breast populations in tissue, their expression pattern may not be as stable in cells in culture [22]. We therefore employ a comprehensive panel of markers to characterise the phenotype of isolated cell populations. Using this combination of markers our findings confirm more than 95% purity of each myoepithelial, luminal and fibroblast population. Specifically, the main contaminants of the stromal component, namely, endothelial cells, pericytes and inflammatory cells were all ruled out on immunofluorescent profiling [23] while the cell populations largely maintained their *in vivo *characteristics. Some differences were noted, particularly down-regulation of α-SMA in the myoepithelial cell cultures, in keeping with other studies [24]. Although primary cells represent the ideal, it is important to attempt to generate viable alternatives, owing to the inherent difficulties of working with this material. We obtained cell lines generated from luminal, myoepithelial and fibroblast populations isolated in a similar fashion to our primary cells and immortalised by transduction with SV40 large T antigen [19]. These cell populations exhibited close phenotypic integrity in comparison to their primary counterparts. In order to incorporate a tumour cell population in the cultures, MCF-7 breast cancer cell line was chosen as a representative, well-characterised luminal-like cell.

Co-aggregation of the epithelial components of the breast resulted in localisation of myoepithelial cells around luminal cells to form a 'co-unit', analogous to the *in vivo *situation. Incorporation of cells into co-cultures without prior co-aggregation led to a similar organisation of myoepithelial cells around the luminal population, suggesting that this is an active 'homing' process. Homing has been shown to be dependant on spatial specificity determined by desmosomal proteins and can be disrupted using peptides to myoepithelial specific desmoglein-3 and desmocollin (Dsc) 3 [25]. In keeping with this, homing was shown to be myoepithelial specific with no evidence of localisation of luminal cells around MCF-7 cells. Similar co-units were generated when either primary myoepithelial cells or the myoepithelial cell line was used supporting the validity of this line. Myoepithelial cells form co-units with both normal and MCF-7 cells but subtle differences were observed between the two culture systems, mainly at the junction of the basal surface of the myoepithelial cells and the basement membrane. In the 'normal' co-units myoepithelial cells were shown to lay down their own basement membrane protein, demonstrated here with TN-C and previously described by other groups in 2D cultures of myoepithelial cells [26]. There was polarised expression of β-integrin located at the basal interface between the myoepithelial cells and the basement membrane in structures resembling hemidesmosomes [26,27]. The most striking difference identified in the presence of a malignant luminal population was disruption of basement membrane and loss of β4-integrin. Basement membrane has been shown to be altered around DCIS [6,27], and loss of hemidesmosomes has also been reported in DCIS tissue [27]. This correlation between the *in vitro *models described here and observations of DCIS clinical samples supports the validity of these co-units as models of normal and DCIS-like breast.

To generate further complexity, fibroblasts, implicated in breast cancer progression, were also incorporated into the model. It is known that TAFs are phenotypically and functionally different to normal fibroblasts; therefore, fibroblasts from normal donors and patients with breast cancer were examined, together with two fibroblast cell lines that reflect 'normal' and 'tumour-like' fibroblasts. Normal fibroblasts homed around the epithelial co-units and did not disrupt the glandular architecture; however, the TAFs led to complete disruption of co-unit organisation. The ability of conditioned media from these fibroblasts to recreate this suggests that the effect is mediated at least in part by soluble factors released from the fibroblasts. Tumour-associated fibroblasts are known to release higher levels of proteases such as urokinase plasminogen activator and MMPs than their normal counterparts [5,28]. Additionally HGF is up-regulated in breast cancer and also released at higher levels by these fibroblasts [2,29].

We therefore used this co-culture model system to investigate the potential involvement of MMPs and HGF signalling in mediating the disruption of normal architecture. The incorporation of either a broad spectrum inhibitor to MMPs or a c-met inhibitor partially abrogated the disruptive effect of TAFs or conditioned medium on co-unit formation. The effect was significantly greater with the MMP inhibitor than with c-met inhibition which suggests that, in this system, MMPs play the more prominent role. Several stromal-derived MMP members have been implicated in playing a role in the transition of DCIS to invasive disease, including MMP-13 [30], MMP-26-mediated activation of MMP-9 [31], and MMP-2, -3 and -11 [32]. The model system described here provides a mechanism to dissect the role of these factors in the control of cellular interactions and tissue architecture.

## Conclusion

The models described here are the first to incorporate not only luminal and fibroblast populations but also the myoepithelial population that *in vivo *forms the interface between the epithelial and stromal compartments. While lacking the full complexity of mouse model systems, this model has several advantages including easy manipulation of individual cell populations, shorter timescale and cost. Furthermore, these models could easily be adapted to incorporate other components of the microenvironment including inflammatory or endothelial cell populations. Of particular importance to the development of human models of cancer, these co-cultures can be set up and maintained in a completely animal-free environment by adapting the reagents used.

In establishing these models, we have identified differences in behaviour between normal and tumour-associated cell populations. These raise important questions about the role of these cell interactions in generating phenotypic and functional changes that may influence tumour behaviour. Our preliminary evidence suggests a role for MMP in mediating the disruption of normal cellular interactions and demonstrates how these models could be used to dissect such interactions, as well as their potential for use in the identification and screening of novel therapeutic targets.

## Abbreviations

3D: three dimensional; BSA: bovine serum albumin; DAPI: 4',6-diamidino-2-phenylindole; DCIS: ductal carcinoma *in situ*; DMEM: Dulbecco's modified eagle's media; DMSO: dimethyl sulfoxide; DSc3: desmocollin-3; ECM: extracellular matrix; EMA: epithelial membrane antigen; FBS: fetal bovine serum; HGF: hepatocyte growth factor; MMP: matrix metalloproteinase; PBS: phosphate buffered saline; SMA: smooth muscle actin; TAF: tumour-associated fibroblast; TN-C: tenascin-C.

## Competing interests

The authors declare that they have no competing interests.

## Authors' contributions

DLH, KTB, AM and LAG carried out all experimental studies. DLH and JLJ designed the study, interpreted the results and drafted the manuscript. All authors read and approved the final manuscript.
